# Notice of Mr. Ring's Unwarrantable Assertion

**Published:** 1816-11

**Authors:** 


THE LONDON

### Medical and Physical Journal.

5 OF VOL. XXXVI.] NOVEMBER, 1816. [NO. 213.

"For many fortunate discoveries in medicine, and for the detection of numerous errors, the world is indebted to the rapid circulation of Monthly Journals; and there never existed any work to which the Faculty in EUROPE and AMERICA were under deeper obligations than to the Medical and Physical Journal of London, now forming a long, but an invaluable, series."—RUSH.

### *For the London Medical and Physical Journal.*

### *Notice of Mr. Ring's unwarrantable Assertion.* By Dr. KINGLAKE.

[How much soever we may feel at misunderstandings among gentlemen of the faculty, it is, at least, some satisfaction to the Conductors of this Journal, that they have not only kept clear of personality themselves, but have usually been selected as the channel by which men of character and abilities have made their appeal to the medical world. They are the more pleased at this, because the circulation of their sheets is principally among those who are competent judges of the questions at issue. Hence artifice and declamation, which might be admitted in more miscellaneous Journals, are not likely to mislead the public.—On this account, we have thought it our duty to pay respect to our valued correspondent, by inserting his communication. It should be remarked, however, that it contains only a vindication of himself, without personal allusion of any kind, excepting as far as relates to expressions which are quoted. On this subject our readers are competent judges. It is ours only to place the naked documents before them.—EDIT.]

I TRUST that it will not be altogether inconsistent with the liberal design of your Journal, to make it the vehicle of contradiction to a most unwarrantable assertion advanced by *a Mr. Ring*, in a recent publication, which he entitles "an Answer to Dr. Kinglake on the Cooling Treatment of Gout." In that production, which has, perhaps, *no parallel* in the records of medical authorship for personal invective and wanton calumny, the fearless author ventures to say, "I am now assured that he (Dr. Kinglake) has never regularly graduated as a physician"! Fraudulently to assume a title is justly held to be amongst the most flagrantly disgraceful offences that can be committed; and to be publicly charged with it, certainly demands the reprehensive and penal notice that it will probably receive in another quarter. In the mean time, I have to request that you will notify my protest against the said Mr. Ring's defamatory attack on my professional reputation; and that you will also submit to the public my refutation of his libellous freedom, by publishing the subjoined copy of my *diploma* of a doctor's degree in medicine, and likewise the title-page of my inaugural dissertation, which, as usual, was published on that occasion.

"*Senatus Universitatis Glasguensis Lectori salutem.*

"Quum vir ornatissimus Robertus Kinglake, A.M. post operam Arti Medicæ sedulo navatam honores a nobis petiverit academicos, nos per universam eum medicinam curavimus examinandum: in quo examine cum præclaram eruditionem et medendi peritiam pari cum modestia conjunctam nobis abunde probaverit et *theses* insuper medicas quas *de Vitæ principio, sanitate et morbo,* scripsit, atque Senatus Academici judicio probatas edidit, publice feliciter propugnaverit: Nos virum ornatissimum summis in medicina honoribus dignum existimavimus. Dictum propterea Robertum Kinglake Medicinæ Doctorem creavimus ac declaravimus et his eum literis Doctorem appellamus, atque apud omnes haberi et appellari volumus; eique potestatem damus plenissimam de re medica, legendi, docendi, consultandi, scribendi et disputandi, in Cathedram Doctoralem ascendendi, omnes denique tam theoriæ quam praxeos actus ubique terrarum exercendi, et omnes simul honores, prærogativas omnes ei concedimus et privilegia, quæ vero Medicinæ Doctori usquam gentium conceduntur aut concedi solent. In quorum fidem literis hisce communi Academiæ sigillo munitis nomina nostra subscripsimus.

*Arch: Davidson, Univers: Vice-Cancel: et Collegii Præfect:*

*Rob: Findlay, S. S. Theol: Prof:*

*Joannes Anderson, Physicæ Prof:*

*Joannes Millar, J. C. P.*

*Pat: Cumin, Litt: Orient: P.*

*Tho: Reid, P. Mor: P.*

*Gul Richardson, L. H. P.*

*H. Macleod, Hist. Profr:*

*Pat: Wilson, Astr: Pract: P.*

*Geo: Jardine, Log: et Rhet: P.*

*Jac: Jeffray, Bot: et Anat: P.*

*Thos: Cars: Hope, Med: Profr:*"

"*Datum Glasguæ*

*Nonis Juniis,*

*Anno Aeræ Christianæ,*

*MDCCXCIV.*"

"Dissertatio

*Title of my inaugural Dissertation.*

"Dissertatio Physiologica Inauguralis, quædam de Vitæ Principio, Sanitate et Morbo complectens: quam, annuente summo numine, ex auctoritate dignissimi Vice-Cancellarii, Archibaldi Davidson, S. S. T. P. P. et Collegii Glasg. Præfecti: nec non amplissimi Senatus Academici consensu, et nobilissimæ Facultatis Medicæ decreto; pro Gradu Doctoris, summisque in Medicina honoribus et privilegiis rite ac legitime consequendis; in Comitiis Universitatis Glasguensis, eruditorum examini subjicit Robertus Kinglake, A.M. Anglus, Societ. Reg. Med. necnon, Societ. Physic. Americ. Edin. Soc. et Lycei Med. Lond. Sod. ad diem v. Junii, hora locoque solitis.

Α'ποκρίνου δὴ ᾧ ἂν τί ἐγενῆται σώματι, ζῶν ἔςαι.
Ω῾ ἂν ψυχη, ἔφη. PLATO.

Nasci ergo de novo nihil, renasci omnia, mutari composita neque interim elementa dissolvi. BOERHAAVE.

Glasguæ: in ædibus Academicis excudebat Andreas Foulis Academiæ Typographus.—M.DCC.XCIV."

---

[We are much concerned to find a worthy and learned character so *tremblingly alive.* At the same time it must be admitted that such a charge, from whatever quarter, cannot pass entirely unnoticed; and we hope it will end in this ample and satisfactory refutation.

We must now make our apology to Dr. Kinglake for what follows. He will first recollect that his last paper arrived late in the month, with, as we fancied, a wish for its early insertion; next, that in a hand-writing with which we are familiar, and which may be called plain, and where there are no technical expressions, we probably trusted to a well-educated reader at the printing-office.—EDIT.]

In my reply to Mr. Atkinson on Obstetric Practice, published in your last Journal, are three typographical errors of importance, in as far as they obscure my *meaning* in one instance, and violate *grammatical concord* in another. The first occurs in page 289, line 22, where, for *inseparably*, read *insuperably;* the second in page 292, line 17, where, for *imputed*, read *imputes;* the third in page 292, line 19, where, for *incapacitates*, read *incapacitate.*

*Taunton; Oct.* 10, 1816.

